# Plakophilin-3 Catenin Associates with the ETV1/ER81 Transcription Factor to Positively Modulate Gene Activity

**DOI:** 10.1371/journal.pone.0086784

**Published:** 2014-01-27

**Authors:** William A. Munoz, Moonsup Lee, Rachel K. Miller, Zamal Ahmed, Hong Ji, Todd M. Link, Gilbert R. Lee, Malgorzata Kloc, John E. Ladbury, Pierre D. McCrea

**Affiliations:** 1 Department of Biochemistry and Molecular Biology, The University of Texas M.D. Anderson Cancer Center, Houston, Texas, United States of America; 2 Program in Genes & Development, The University of Texas Graduate School of Biomedical Science - Houston, Texas, United States of America; 3 Center for Biomolecular Structure and Function, The University of Texas M.D. Anderson Cancer Center, Houston, Texas, United States of America; 4 Department of Surgery, Houston Methodist, Houston Methodist Research Institute, Houston, Texas, United States of America; Northwestern University Feinberg School of Medicine, United States of America

## Abstract

Members of the plakophilin-catenin sub-family (Pkp-1, -2, and -3) facilitate the linkage of desmosome junctional components to each other (e.g. desmosomal cadherins to desmoplakin) and the intermediate-filament cytoskeleton. Pkps also contribute to desmosomal stabilization and the trafficking of its components. The functions of Pkps outside of the desmosome are less well studied, despite evidence suggesting their roles in mRNA regulation, small-GTPase modulation (e.g. mid-body scission) during cell division, and cell survival following DNA damage. Pkp-catenins are further believed to have roles in the nucleus given their nuclear localization in some contexts and the known nuclear roles of structurally related catenins, such as beta-catenin and p120-catenin. Further, Pkp-catenin activities in the nuclear compartment have become of increased interest with the identification of interactions between Pkp2-catenin and RNA Pol III and Pkp1 with single-stranded DNA. Consistent with earlier reports suggesting possible nuclear roles in development, we previously demonstrated prominent nuclear localization of Pkp3 in *Xenopus* naïve ectoderm (“animal cap”) cells and recently resolved a similar localization in mouse embryonic stem cells. Here, we report the association and positive functional interaction of Pkp3 with a transcription factor, Ets variant gene 1 (ETV1), which has critical roles in neural development and prominent roles in human genetic disease. Our results are the first to report the interaction of a sequence-specific transcription factor with any Pkp. Using *Xenopus laevis* embryos and mammalian cells, we provide evidence for the Pkp3:ETV1 complex on both biochemical and functional levels.

## Introduction

Plakophilins (Pkps) form a subfamily of catenins, each containing an Armadillo-repeat domain that is structurally homologous to those present in members of the beta-catenin and p120-catenin subfamilies [1]–[6]. Pkps assist in assembling and stabilizing desmosomal cell-cell junctions through interactions with the cytoplasmic tail of desmosomal cadherins (desmocollins and desmogleins). Such cadherins span the plasma membrane and associate with intermediate filaments through interactions with intracellular desmosomal components such as desmoplakin [3], [7]–[10]. These interactions promote the physical integrity of multiple tissues subject to mechanical stress [8], [11]–[19]. While their cytoplasmic functions are less understood, Pkp-catenins are involved in regulating mRNA expression, the trafficking of desmosomal cadherins, and insulin-induced cell proliferation [20]–[23]. In addition to such junctional and cytoplasmic localizations and roles, Pkp-catenins were previously reported in the nucleus in some contexts [6], [24]–[31]. Conceivably, some Pkp-catenin roles might later prove to be analogous to those of the beta- and p120-catenin subfamily members that associate directly with transcription-factors (e.g. beta-catenin binds/modulates TCF/LEF, and p120-catenin binds/modulates Kaiso) [32]–[34]. As published thus far, Pkp nuclear associations have intriguingly included Pkp1-catenin binding to single-strand DNA and Pkp2-catenin to the RNA polymerase III holoenzyme [26], [35]. The functional roles of these nuclear interactions still require clarification.

In this study, we show for the first time to our knowledge that a Pkp-catenin specifically interacts with a transcription factor. We reveal that Pkp3-catenin binds to ETV1, a member of the larger E-twenty six (Ets) family whose members bind DNA directly (e.g. via a conserved Ets domain) (reviewed in Hollenhorst et al., 2011) [36]. By virtue of their roles in gene control, Ets-members have been found to exhibit key roles in vertebrate and invertebrate development and human disease [37]–[39]. In particular, ETV1 is known to contribute to the formation of dopaminergic neurons through the regulation of various dopamine transport and synthesis genes and also contributes to formation of proper connections between group 1a sensory afferents and motor neurons [38], [39]. In pathology, ETV1 functions in Ewing’s sarcomas through a fusion with the Ewing’s sarcoma gene (Ews) and promotes the metastasis of prostate cancer (reviewed in Oh et al., 2012) [40]. Our work employs both *Xenopus laevis* embryos and mammalian cell-line based assays and supports the interaction of Pkp3-catenin with ETV1 on both biochemical and functional grounds. We further find that Pkp3-catenin associates with ETV5, which is closely related to ETV1 and belongs to the same three-member Polyomavirus enhancer activator 3 (PEA3)-subgroup of Ets-family members. Altogether, we report the first interaction of a plakophilin-catenin with a site-specific DNA-binding transcription factor, namely Pkp3-catenin’s association with ETV1. In providing an initial analysis of their nuclear relationship, this work should assist in furthering an understanding of how Pkp-catenins influence development and possibly disease.

## Materials and Methods

### Embryo Manipulations and Ethics

*Xenopus laevis* embryos were obtained, fertilized and microinjected as previously described [24], [41]. This study was carried out in strict accordance with recommendations in the American Association for Laboratory Animal Science Learning Library. The protocol was approved by the Institutional Animal Care and Use Committee of The University of Texas-M.D. Anderson Cancer Center (ACUF Protocol #09-93-05717). All efforts were made to minimize *Xenopus laevis* suffering.

### Yeast Two-hybrid

Full-length *Xenopus* Pkp3 was used to screen an adult mouse brain cDNA library in collaboration with Hybrigenics ULTImate Y2H [42]. We obtained 351 positive clones that were then sequenced and analyzed. We were attracted to two clones in particular, ETV1/ER81 and ETV5/ERM), given that they fall within the same PEA subgroup of Ets-family members, which are known to have wide ranging roles in transcriptional control [40], [43], [44].

### Nuclear Fractionation, Immunoprecipitation, and Immunoblotting

Nuclear fractionation of mouse embryonic stem cells required a 10 cm plate of AB-1 wild type cells at 80% confluence for 3 immuno-precipitating conditions. One plate of cells was collected by scraping, washed with 10 mL PBS and pelleted by centrifugation at 200×g for 2 minutes. The pellet was resuspended by gentle pipetting in 600 ul ice-cold buffer A (20 mM HEPES, pH 7.9; 10 mM KCl; 0.2 mM EDTA; protease inhibitors). The cells are allowed to swell for 15 minutes on ice. Following, 37.5 ul of 10% NP-40 was added and the tube vortexed 10 seconds. Nuclei were collected by centrifugation at 200×g for 10 minutes (supernatant contains cytoplasm and the pelleted nuclei), and the nuclei were resuspended in 300 ul ice-cold buffer C1 (20 mM HEPES, pH 7.9; 400 mM NaCl; 10 mM EDTA; 1 mM EGTA; protease inhibitors) and were rocked at 4°C for 1 hour, prior to centrifugation at 16,100×g (microfuge maximum speed) at 4°C for 10 minutes (supernatant contains the nuclear extract). For immuno-precipitations, extracts were transferred to new tubes and diluted with one volume of ice-cold buffer C2 (20 mM HEPES, pH 7.9; 10 mM EDTA; 1 mM EGTA; protease inhibitors). One aliquot was set aside for whole nuclear lysate detection. Extract was diluted to a final volume of 1.5 mL with cold buffer C3 (20 mM HEPES, ph 7.9; 200 mM NaCl; 10 mM EDTA; 1 mM EGTA; protease inhibitors).

Protein A/G PLUS-Agarose (Santa Cruz Biotechnology SC-2003) (10 ul packed agarose/condition) was washed three times in cold buffer C3. After the last wash, the precipitating antibody was added to the beads (e.g. anti-Pkp3 hybridoma supernatant 11F2–300 uL; anti-ETV1–3 ug; mouse IgG –3 ug) and was rocked 45 minutes at 4°C. The beads were centrifuged at 1,000×g for 2 minutes and washed 3× with cold buffer C3 containing 0.5% Triton X-100.

Following agarose bead preparation and nuclear lysate dilutions, 500 uL of lysate was added to the beads and rocked gently for 1 hour at 4°C, centrifuged at 1,000×g for 2 minutes, and washed 3× with cold wash buffer (20 mM HEPES, ph 7.9; 200 mM NaCl; 10 mM EDTA; 1 mM EGTA; 0.5% Triton X-100; 0.5% sodium deoxycholate; 0.1% SDS). The final bead pellet was resuspended in 30 ul of 2× protein sample buffer and the entire sample loaded. Immunoblotting was carried out following standard protocols.

### RNA Isolation and Semi-quantitative Real Time PCR

RNA isolation and semi-quantitative real time PCR were performed as previously described [24]. Briefly, total RNA was extracted from *Xenopus* embryos using Trizol (Invitrogen). DNA was removed with RQ1 DNase (Promega M610A). Constant amounts of total RNA were reverse-transcribed into cDNA pools using oligo-dT (Invitrogen 18418-012) and SuperScript III Reverse Transcriptase (Invitrogen 18080-044). cDNA was then used as template for either real time PCR with SYBR Green Master Mix or PCR amplification. To control for DNA contamination, reactions were performed in the absence of reverse transcriptase. PCR primers are as follows: DDC 5′-TGGAGAGGGCTGGACTGATC-3′, DDC-R 5′-AGCCTGACCACGAGCTACAAA-3′, TH-F 5′-GCCGGATTGTTGCCATGA-3′, TH-R 5′-GGCCCCCAAAGATGCTAAAC-3′, SLC18A2-F 5′-GCCCCCCGCTATCAACTT-3′, SLC18A2-R 5′-ACCTTCCGATTTTTTGTGCAA-3′, GCH-F 5′-TCTGGTGTTGGAGTGGTTGTTG-3′, GCH-R 5′-CCCCTAGCATGGTACTCGTTACA-3′ ODC-F 5′-AAAAAGCATGTGCGTTGGT-3′, ODC-R 5′-ACGGCATAAAACGGAGTGA-3′.

### Whole-mount in situ RNA Hybridization

Whole-mount in situ RNA hybridization was performed as previously described [45]. Briefly, digoxigenin-labeled sense and antisense RNA probes were prepared through in vitro transcription (DIG RNA Labeling Kit, Roche 11175025910). Probes were detected with anti-digoxigenin antibody conjugated to alkaline phosphatase (Roche 11093274910) and NBT/BCIP (Roche 11697471001) was used as the substrate during reactions.

### Antibodies

Anti-human Pkp3 hybridoma supernatant clone 11F2 was kindly provided by Dr. JK Wahl, 3^rd^. All other antibodies were purchased from commercial suppliers (mouse anti-c-myc, DSHB 9E 10; rabbit anti-myc, Santa Cruz SC-789; mouse anti-HA, 12CA5; rabbit anti-HA Santa Cruz SC-805; ETV1, Thermo Scientific MA5-15461; Pkp3, Progen 651113). Secondary antibodies were Alexa Fluor 488 labeled goat anti-rabbit antibody (Invitrogen), Alexa Fluor 555 labeled goat anti-mouse antibody (Invitrogen), HRP-conjugated goat anti-rabbit IgG (Bio-Rad) and HRP-conjugated goat anti-mouse IgG (Bio-Rad).

### Cell Culture

Human HeLa and HEK 293T cells were cultured in DMEM (Invitrogen), using standard mammalian cell culture conditions [46]. Cells were transfected using either Lipofectamine-2000 (Invitrogen) or polyethylenimine following manufacturer’s instructions. Mouse wild type AB-1 embryonic stem cells were cultured as previously described [47].

### Immunofluorescence

HeLa cells were plated on glass coverslips and grown to either 60 or 90% confluence 24 hours post-transfection. Cells were washed three times in 1X PBS at room temperature (RT) and then fixed in PBS with 4% paraformaldehyde for 10 minutes at four degrees Celsius. Cells were then washed three times in 1X PBS at RT, before neutralizing the paraformaldehyde with 50 mM NH4Cl for 10 minutes at RT. Following two additional washes with 1X PBS, the cells were permeabilized with 0.5% Triton X-100 in PBS for 10 minutes at RT. Two 1X PBS washes preceded a one-hour RT incubation in blocking buffer (10% goat serum, 2 mg/ml BSA, 0.1% Triton X-100 in 1X PBS). Cells were then incubated overnight at four degrees Celsius in blocking buffer containing primary antibody (1∶500 dilutions of either anti-HA or anti-Myc). The next day, samples were washed 3X for 10 minutes each in 1X PBS. They were then incubated for 1 hour at RT with Alexa Flour® 488 or 555 conjugated secondary antibodies (1∶500). Three washes were performed prior to mounting with Vectashield® mounting medium (Vector Laboratories Cat. No. H-1000).

Images were obtained with a Zeiss Observer Z1 confocal designed by 3i, Inc using Slidebook 5.5 software. To ensure negative interactions exhibited no relocalization, images were adjusted for brightness in select cases using Adobe Photoshop software.

### Antisense Morpholinos

*Xenopus* Pkp3 morpholino (Pkp3 MO 1) and standard control morpholino (SC MO) were obtained and used as previously described [24].

### Plasmid Constructs and in vitro Transcription

Using standard recombinant techniques as previously described [46], pCS2-based plasmids were made encoding *Xenopus* ETV1 (or ETV1 mapping regions) fused to an amino-terminal Myc-tag, while *Xenopus* Pkp3 was fused to an amino-terminal HA-tag or to a Bcl-Xl MOM targeting sequence. Production of in vitro transcribed RNA was done following previously described methods [24]. Other constructs were reported previously and kindly provided by Dr. L. Shemshedini (Fes3xWT-Luc and MMP1-Luc) and Dr. R. Janknecht (MMP1 −87/−88) [48]–[50].

### Luciferase Assays and Cell-free Transcription-translation (TnT) System

HEK 293T cells were co-transfected with the indicated plasmids and pRL-SV40 renilla reporter plasmid (internal control). Cells were lysed and assayed 24 hours later for luciferase activity using the Dual Luciferase® Reporter Assay System, in accordance with the manufacturer’s protocol (Promega). All experiments were repeated a minimum of three times.

For cell-free transcription-translation, xPkp3-HA or xETV1-Myc were transcribed and translated using the TnT® Sp6 high-yield wheat germ protein expression system (Promega Cat. No. L3260) following the manufacturer’s protocol. Translated protein was then added to a second TnT® Sp6 high-yield wheat germ reaction containing the Fes3xWT-Luc construct and incubated for 2 hours at 25°C. 5 uL of each reaction was diluted in 15 uL of 1X lysis buffer and tested according to the Dual Luciferase® Reporter Assay System, in accordance with the manufacturer’s protocol (Promega).

### Statistical Analysis

Phenotypic analysis data is presented as percentages to account for differences in the total numbers obtained between each experimental condition. Overall, graphs are based on the average percentages of triplicate experiments. The Student’s T-test was used to analyze the significance of observed phenotypes. To obtain P-values, we hypothesized that the two groups are statistically identical (null hypothesis), and analyzed the data using the Student’s T-test (TTEST) within the Microsoft Excel software program.

## Results

### Yeast Two-hybrid Screening Identifies a Novel Pkp3-associated Protein

**Figure 1:**
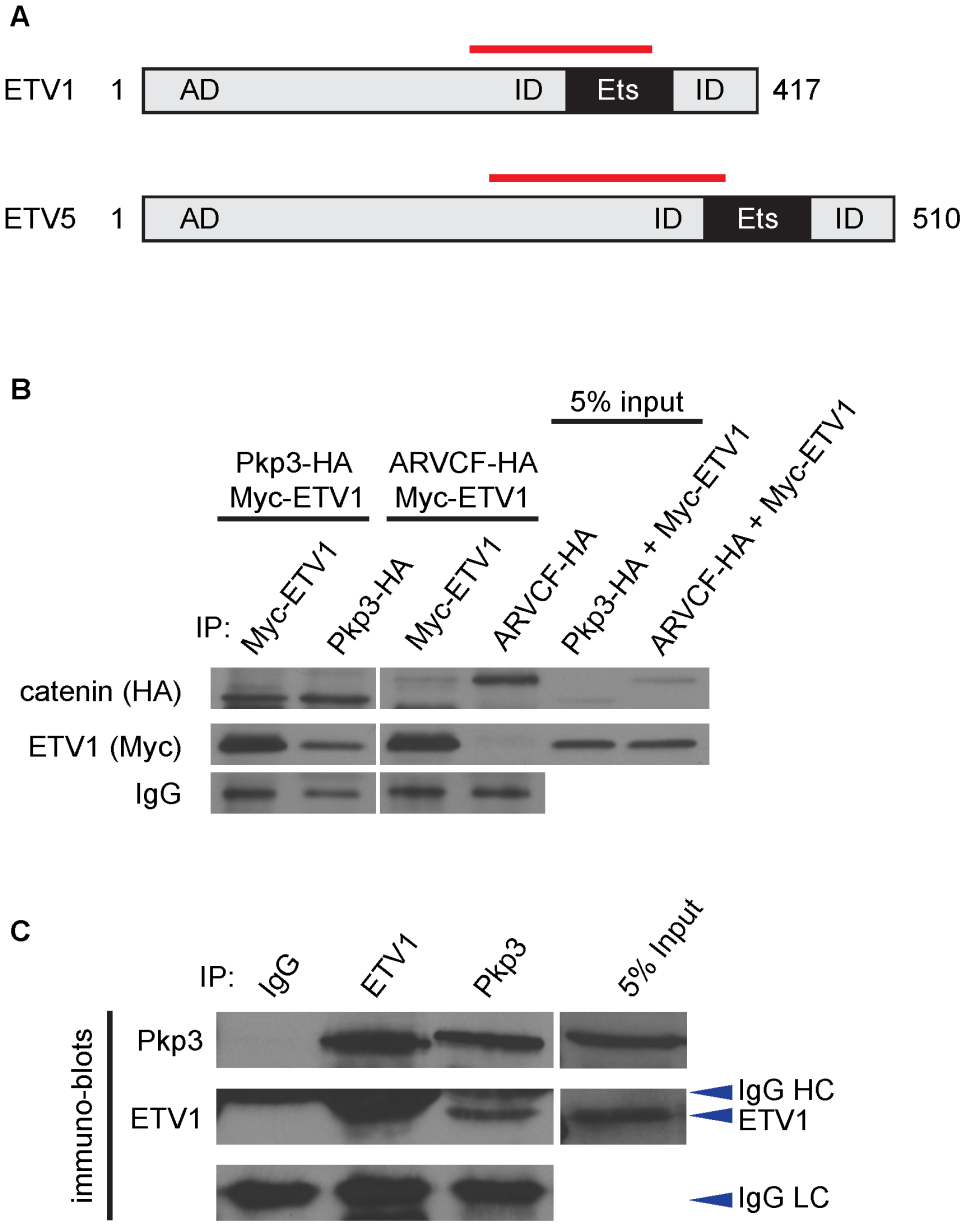
ETV1 is a novel binding partner of Pkp3-catenin. (**A**) Schematics of ETV1 and ETV5 protein structures. Red line indicates the binding region as suggested by sequenced prey clones identified from yeast-two hybrid screen. (**B**) Either HA-Pkp3 or HA-ARVCF was co-transfected with Myc-ETV1 in HEK 293T cells. Anti-Myc (ETV1) or –HA (Pkp3 or ARVCF) immune-precipitates were subjected to immuno-blotting with anti-Myc (ETV1) and –HA (Pkp3 or ARVCF) antibodies. Pkp3-catenin co-precipitates ETV1, but the structurally homologous ARVCF-catenin does not (negative/specificity control). ETV1 co-precipitates Pkp3, but does not co-precipitate ARVCF. (**C**) Untreated AB-1 wild type mouse embryonic stem cells were lysed and fractionated at 90% confluency and endogenous Pkp3 or ETV1 was immuno-precipitated from nuclear extracts, followed by immuno-blotting for Pkp3 and ETV1. Five percent inputs were acquired separately due to the low levels of endogenous proteins.

Given the strong nuclear localization of *Xenopus laevis* Pkp3 in naïve ectoderm (“animal cap”) cells and Pkp3’s suggested functional roles in *Xenopus* neural development described previously [24], we aimed to identify novel nuclear binding partners of Pkp3 involved in neural development. Employing *Xenopus laevis* Pkp3 as bait, we performed a yeast two-hybrid screen using an adult mouse brain cDNA library (Hybrigenics, Inc.). 351 clones were obtained, representing 156 distinct potential interactions. This screen suggested that Pkp3 has roles in varied cellular compartments, with potential partners contributing to cell shape, intracellular trafficking, small-GTPase and cell cycle activity, along with RNA processing and gene transcription. Included among the transcriptional regulators were ETV1 and ETV5, members of the large DNA-binding Ets-family falling within the much smaller (3-member) PEA3 sub-family. Clones of ETV1 and ETV5 identified in the screen suggested that homologous regions interact with Pkp3. The region identified is proximal to and partially includes the Ets/DNA-binding domain (Figure 1A). We chose to further test the Pkp3:ETV1 interaction, given that each gene-product has a similar spatial expression in *Xenopus* [24], [51] and the existence of evidence that each contributes to neural development [39], [52], [53]. Our yeast two-hybrid screen of mouse brain was not saturating, as the desmocollin and desmoglein desmosomal-cadherins as well as previously identified RNA binding partners were not isolated [2], [3], [8], [11], [12], [20], [54]. The absence of the former is likely due to the low expression of desmosomal components in neural structures following neurulation [55]. Interestingly, β2-spectrin, an ARVCF-catenin binding protein [46], was a strong candidate for interaction with Pkp3. This might indicate shared functional association of these catenins at cortical regions, requiring later investigation. For our purposes here, the structural homology between Pkp3- and ARVCF-catenin especially within the Armadillo domain has provided a robust negative control (ARVCF) to investigate the specificity of Pkp3 binding to Etv1. In summary, we report that a prominent member of the PEA3 subgroup of Ets-family transcription factors, Etv1 (and also Etv5), binds and functionally interacts with the Pkp3-catenin.

### ETV1 Interacts with Pkp3-catenin in Cells

We first sought to confirm the Pkp3:ETV1 interaction using exogenously expressed *Xenopus* proteins. Antibodies are not available to precipitate endogenous *Xenopus* Pkp3 or ETV1, so HA-tagged xPkp3 (or the negative control xARVCF) was first co-expressed with Myc-tagged xETV1 in mammalian HEK 293T cells. HEK 293Ts cells provide the advantage of lacking detectable endogenous Pkp3 or ETV1 that could compete for exogenous interactions. In agreement with our yeast two-hybrid screen, the immuno-precipitation of HA-xPkp3 indicated that it interacts with Myc-xETV1, as did the reverse immuno-precipitation of Myc-xETV1 (Figure 1B, lanes 1 and 2). Suggesting the specificity of the Pkp3:ETV1 interaction, ARVCF-catenin, which is structurally related to Pkp3-catenin, did not or only very weakly associated with Myc-xETV1 (Figure 1B, lanes 3 and 4).

To test the conservation of the interaction across vertebrate species (*Xenopus* versus mouse) and confirm a nuclear endogenous interaction in a more developmental context, we turned to the use of mouse embryonic stem cells (mESCs). mESCs exhibit easily-detectible endogenous expression of Pkp3 and ETV1 (Figure 1C), and we observed Pkp3’s prominent presence in the nucleus following cell fractionation (Figure S1A). Importantly, co-immunoprecipitation assays from nuclear lysates in this endogenous context further indicated that Pkp3 associates robustly with ETV1 in the nucleus (Figure 1C), whereas IgG control precipitations failed to resolve co-associations as expected. In HeLa cells, faint but reproducible nuclear localization of Pkp3 was also observed via immuno-fluorescent localization following the exogenous co-expression of Pkp3 and ETV1 (Figure S1B). In summary, as had been suggested from yeast two-hybrid screening, our initial biochemical results supported the authenticity of the Pkp3-catenin:ETV1 association.

### Interaction Domain Mapping of the Pkp3:ETV1 Complex

**Figure 2:**
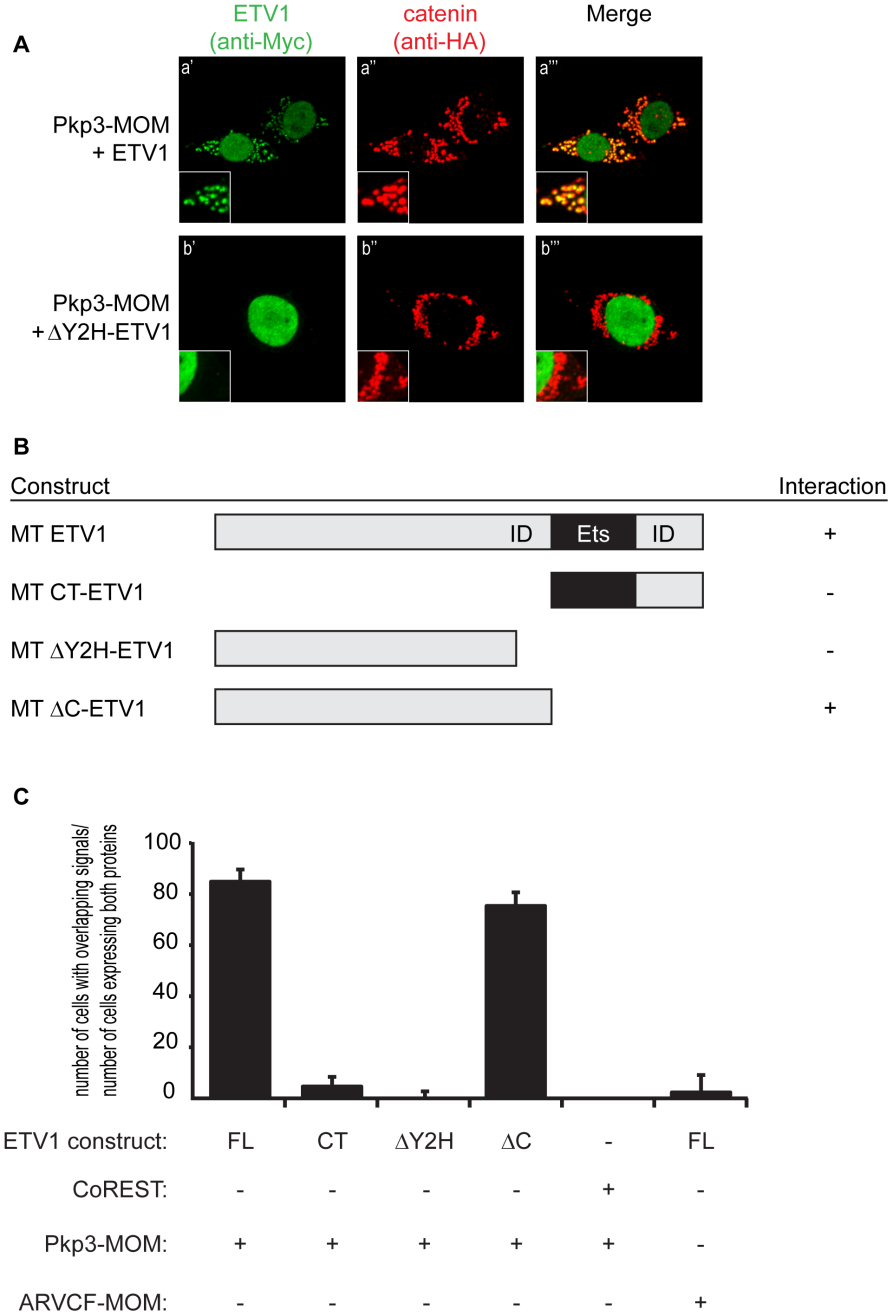
Ectopic mitochondrial outer membrane (MOM) co-relocalization in HeLa cells of xPkp3 with xETV1, to map ETV1’s binding domains. (**A**) Co-expression of Myc-xETV1 with MOM-targeted HA-xPkp3. Cells were co-immunostained for the ectopic co-relocalization of xETV1 (Myc-epitope, left panels), as well as for the MOM-targeted catenin (HA-epitope, middle panels). Ectopic co-relocalization of xETV1 with MOM-targeted xPkp3 was only observed when amino acids 257–335 of xETV1 were present. Representative images from a minimum of sixty cells expressing both constructs analyzed are shown. (**B**) List of Myc-tagged (MT) xETV1 constructs and their interaction status with MOM-targeted HA-xPkp3. A positive score (+) indicates that >60% of cells coexpressing the indicated xETV1 deletion construct showed co-relocalization of xETV1 with the MOM-targeted HA-xPkp3 construct. Our prior laboratory studies have shown that authentic partner proteins re-localize to the MOM (fusion protein), as visualized by their increased signal at the mitochondria [46]. (**C**) Quantification of the extent of signal overlap from MOM-targeted xPkp3 or MOM-targeted xARVCF with the indicated xETV1 mapping constructs. Minimally 30 cells from each replicate experiment (totaling over 100 cells from triplicate experiments) were quantitated for the extent of signal overlap.

To identify the domain(s) of ETV1 that interact(s) with Pkp3, an assay that targeted Pkp3 ectopically to the mitochondrial outer membrane (MOM) was utilized in conjunction with deletion constructs of ETV1. The MOM localization domain of Bcl-X_L_ was carboxy-terminally fused to xPkp3-catenin (or to negative-control xARVCF-catenin), as previously undertaken for p120-catenin [46]. These fusion proteins localize to the MOM in a punctate pattern when expressed in HeLa cells, which were used since they facilitate visualization (Figure S2; similar results were obtained using HEK 293T cells; data not shown). Four xETV1 deletion constructs expressed at similar levels were tested for interaction with xPkp3-MOM (Figure 2A, S3A and data not shown). While the signals of the relocalized Myc-ETV1 constructs were faint, we found, as expected, that full-length ETV1 reproducibly relocalized in part to the mitochondrial outer membrane when co-expressed with xPkp3-MOM but not when co-expressed with the structurally homologous xARVCF-MOM, demonstrating specificity (Figure 2Aa versus S3Ad). Our yeast two-hybrid analysis had suggested that a proximal part of the ETS domain, plus a putative inhibitory region immediately upstream in the protein sequence [36], was necessary for the xPkp3:xETV1 interaction. Deletion of this proximal inhibitory region in ETV1 (constructs CT-ETV1 or ΔY2H-ETV1), abolished xETV1’s recruitment to xPkp3-MOM (Figures 2Ab and S3Aa). Even as the general distribution of CT-ETV1 initially led to the appearance of an interaction (signal overlap) with xPkp3-MOM, examination of individual optical sections showed that CT-ETV1 was not present at sites of Pkp3-MOM (Figure S3B). Interestingly, deletion of the ETS domain and C-terminus of ETV1 (ΔC-ETV1) has no effect on ETV1’s ability to relocalize with xPkp3-MOM. Consistent with suggestions from our initial yeast two-hybrid screen, these data point to a region of xETV1 necessary for interaction with xPkp3 that includes the proposed inhibitory domain (amino acid residues 257–335).

**Figure 3:**
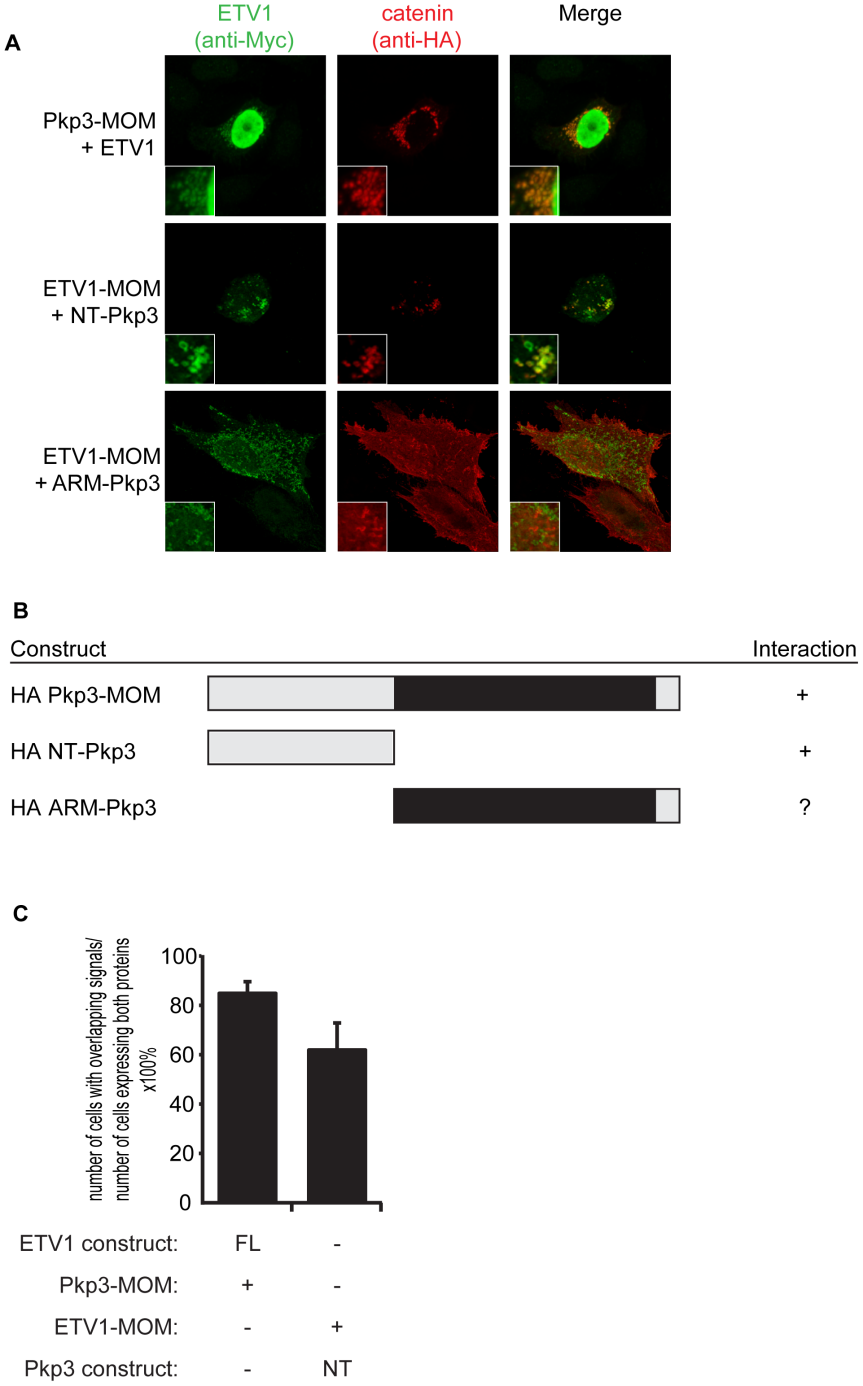
Ectopic mitochondrial outer membrane (MOM) co-relocalization in HeLa cells of xETV1 with xPkp3, to map Pkp3’s binding domains. (**A**) Co-expression of HA-xPkp3 deletion constructs with MOM-targeted Myc-xETV1, or co-expression of Myc-xETV1 with MOM-targeted HA-xPkp3. Cells were co-immunostained for the ectopic co-relocalization of xETV1 (Myc-epitope, left panels), as well as for xPkp3 (HA-epitope, middle panels). Ectopic co-relocalization of xPkp3 with MOM-targeted xETV1 was observed when amino acids 1–325 of xPkp3 were present, while co-relocalization of xPkp3’s ARM domain was inconclusive due to its broad expression. Shown are representative images from a minimum of sixty cells that expressed both constructs and were analyzed. (**B**) List of HA-tagged (HA) xPkp3 constructs, and their interaction status with MOM-targeted Myc-xETV1 (or the inverse). A positive score (+) indicates that >60% of cells coexpressing the indicated xPkp3 deletion construct showed its co-relocalization with MOM-targeted Myc-xETV1. (**C**) Quantification of cells showing overlapping relocalization of MOM-targeted xPkp3 or xARVCF and xETV1 mapping constructs from Fig. 3A. Minimally 30 cells from each replicate experiment (totaling over 100 cells from triplicate experiments) were quantitated for their ability to co-relocalize.

Conversely, we used a construct of xETV1 targeted to the mitochondrial outer membrane (xETV1-MOM) to map Pkp3’s interaction-domain with Etv1. We found that Pkp3’s amino-terminal region is sufficient for the association (Figure 3). It is less clear whether Pkp3’s Armadillo domain might also be involved, given that its localization with ETV1-MOM is weaker than the amino-terminal region of Pkp3, yet a faint co-localized signal is evident.

### xETV1 Partially Rescues Molecular Phenotypes Resulting from xPkp3 Depletion in *Xenopus* Embryos

**Figure 4:**
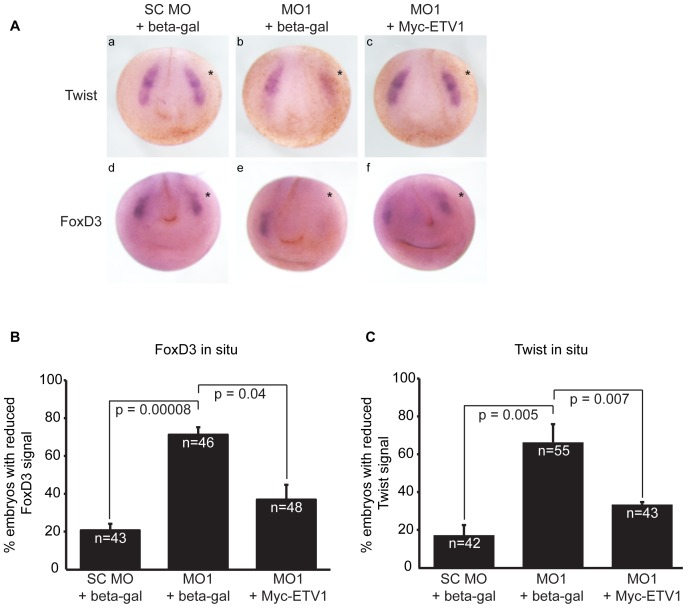
Rescue of neural crest establishment defects, induced upon xPkp3-catenin depletion, through ectopic expression of xETV1. (**A**) The indicated morpholinos (20 ng) and mRNAs (0.5 ng) were co-injected into one dorsal blastomere at the four-cell stage with rhodamine dextran as a lineage tracer. After in situ hybridization for neural crest markers, Twist and FoxD3, embryos were imaged. The ectopic expression of ETV1-Myc largely rescued Twist and FoxD3 expression in embryos depleted for Pkp3-catenin (compare right-most images to middle images). (**B**) Quantification of embryos processed for Twist in situ hybrization, comparing the uninjected side to the injected side, indicated by an asterisk, for reduction in signal. (**C**) Quantification of embryos processed for FoxD3 in situ hybrization, comparing the uninjected side to the injected side for reduction in signal. P-values indicate statistical significance.

We previously reported the morpholino-directed depletion of Pkp3 (>60% efficiency as shown in Figure 5B of Munoz et al., 2012) in early *Xenopus laevis* embryos results in altered neural crest establishment, as well as ectodermal fragility, touch hyposensitivity and heart defects [24]. Additionally, it has been reported that ETV1 localizes to neural crest cells during development and that other PEA3-subfamily members have roles in crest formation [51], [56]. To test for an in vivo functional interaction between xPkp3 and xETV1, we attempted to rescue knock-down phenotypes following xPkp3 depletion, through the exogenous expression of xETV1. Given our earlier work demonstrating that neural crest defects arise following xPkp3 knockdown [24], we focused on general markers of neural crest establishment that are not direct targets of ETV1, namely Twist and FoxD3 (Figure 4). As shown in Figure 4B and 4C, xPkp3 depletion resulted in a reduction in Twist and FoxD3 expression as expected. This xPkp3 depletion effect was significantly rescued upon the coordinate ectopic expression of xETV1. In agreement with findings presented below that were obtained using mammalian cell lines, these results are consistent with xPkp3 and xETV1 interacting positively at the functional level in vivo (embryos), in keeping with their noted biochemical association.

**Figure 5:**
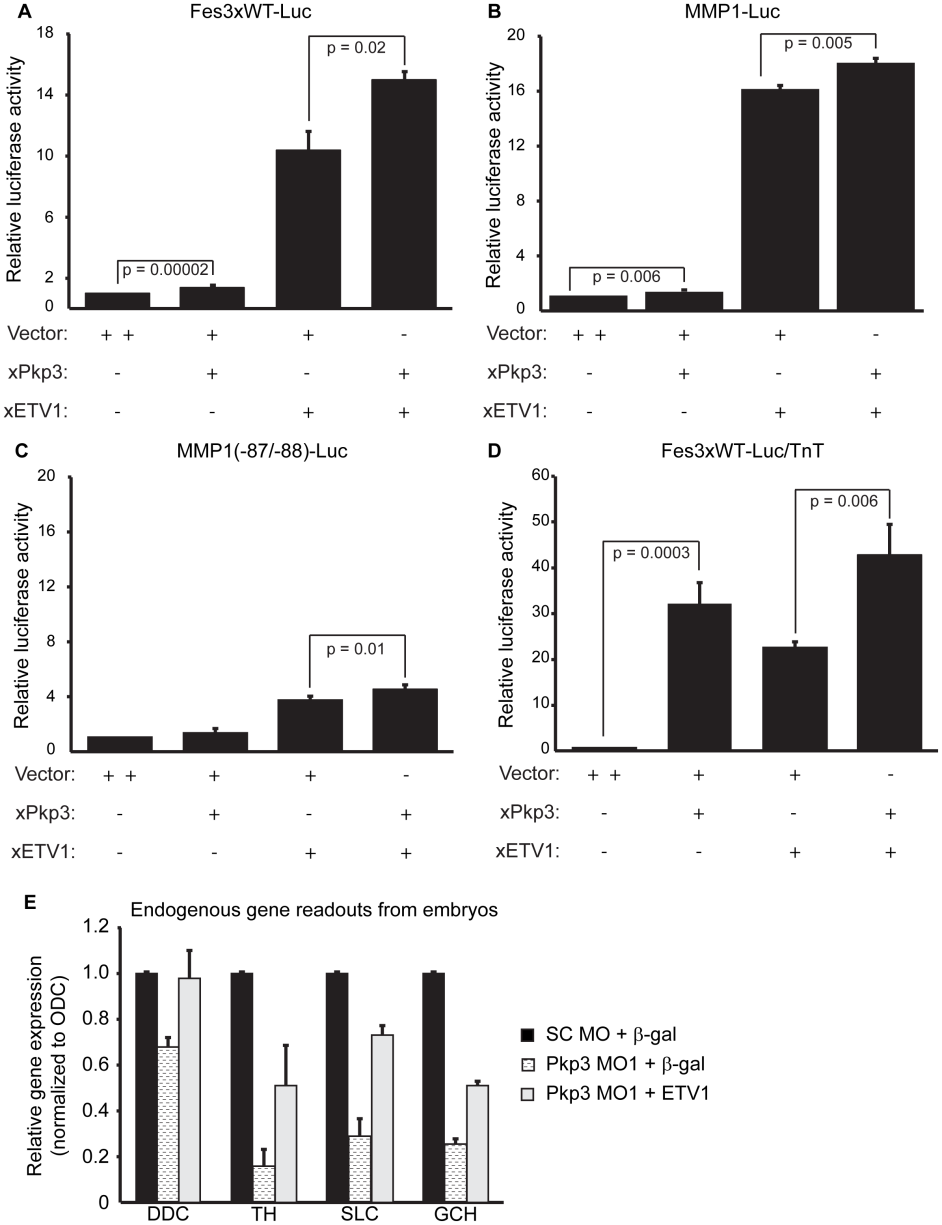
Pkp3-catenin regulation of ETV1 reporters or target genes. HEK 293T cells were transfected with expression plasmids for Myc-xETV1 or HA-xPkp3 in the indicated combinations. (**A**) Pkp3’s functional relationship with ETV1 was tested using an established luciferase reporter for Ets family member transcriptional activity harboring a *fes* gene promoter fragment in triplicate (Fes3xWT-Luc, [57]). Labelled p-values indicate changes that are statistically significant. (**B**) Pkp3’s functional relationship with ETV1 was tested using a second established ETV1 reporter construct harboring a native matrix metalloproteinase-1 (MMP1) promoter fragment (MMP1-Luc,[48]–[50]). (**C**) MMP1(−87/−88)-Luc is a negative-control construct containing a non-functional ETV1 consensus binding site. Labelled p-values indicate changes that are statistical significant. Performed in parallel to Figure 4B. (**D**) To assess Pkp3 and ETV1 activity in a cell-free (in vitro) context, we use the Fes3xWT-Luc reporter and in vitro translated the ETV1 and Pkp3 proteins (TnT Wheat Germ Extract). (**E**) The indicated morpholinos (40 ng) and mRNAs (0.5 ng) were injected into one-cell (cleavage) stage *Xenopus* embryos followed by RNA extraction at late neurula stages, DNAse treatment, reverse transcription, and real-time PCR to assess Pkp3- depletion effects upon ETV1 target-gene transcription. The co-injection of ETV1 mRNA with Pkp3 morpholino (MO) significantly rescued the impact of Pkp3-catenin depletion. The ETV1 gene targets assessed are components of the dopamine synthesis and transport pathways and include: DDC = dopa decarboxylase; TH = tyrosine hydroxylase; SLC = solute carrier family 18 member 2; and GCH = GTP cyclohydrolase I. All real-time PCR was repeated more than three times with samples tested in triplicate with similar outcomes. Error bars represent the standard deviation from three independent experiments. Differences between negative control (SC MO+β-gal) and Pkp3 knockdown (Pkp3 MO1+ β-gal) are all statistically significant based on the Student’s T-Test, demonstrating p-values <0.05. Differences between Pkp3 knockdown (Pkp3 MO1+ β-gal) and its rescue with ectopic ETV1 (Pkp3 MO1+ ETV1) are all statistically significant based on the Student’s T-Test, demonstrating p-values <0.05.

### Pkp3 Positively Modulates ETV1-dependent Transcriptional Activation

Based upon the in vivo rescuing activity of ETV1 following Pkp3 depletion (see above), we expected that ETV1 and Pkp3 might positively interact to regulate gene activity. We first addressed this possibility of using an established artificial ETV1 reporter plasmid containing a *fes* gene promoter fragment encoding the ETV1 consensus sequence (CAGGAAAG), repeated in triplicate (Fes3xWT-Luc) [43], [48], [57]. As shown in Figure 5A, xPkp3 transfection alone resulted in a subtle effect upon the reporter activity (compare bars 1&2). This modest effect is significant (p<0.05) and might result from xPkp3 binding to endogenous ETV1 (although ETV1 is not detected in immuno-blots) bound to the Fes3xWT-Luc reporter. More obvious is xPkp3’s positive impact when exogenous ETV1 is co-expressed (compare bars 3&4). A less obvious but still statistically significant effect (p = 0.005) was reproducibly observed when assessing an independent natural reporter of ETV1 activity, which is based upon a promoter control-element of matrix metalloproteinase-1 (MMP1-Luc; Figure 5B). To test if endogenous ETV1 might contribute to exogenous Pkp3’s ability to activate these reporters, we used a mutant of MMP1-Luc that is no longer recognized by ETV1 [50]. The MMP1 promoter mutation is largely effective, as is reflected in the different relative activation levels of the native versus mutant constructs following ETV1 expression (bar 3 in Figures 5B versus 5C). Note that the data for Figures 5B and 5C were obtained at the same time but were separated graphically to assist with visualization. With the mutant MMP1 luciferase construct, exogenous xPkp3 no longer significantly induced promoter activity by itself (Figure 5C, compare bars 1&2, p = 0.2), and only weakly enhanced an already weak ETV1 activation (compare bars 3&4). This suggests that xPkp3’s observed effects are largely dependent on ETV1’s ability to bind to the promoter of MMP1, and possibly, the minimal reporter activation that arises upon xPkp3’s isolated transfection is due to the subtle presence of endogenous ETV1 or possibly of the related ETV5.

Of note and requiring future investigation, Pkp3 alone was able to activate the Fes3xWT-Luc reporter when its activity was probed using a cell-free in vitro/transcription-translation system (Figure 5D). Since Pkp2 is reported to bind RNA polymerase III [35], it is conceivable that Pkp3 may exhibit analogous interactions that promote reporter activity in this in vitro context. In any case, relative to ETV1 alone, we observed in a cell-free (in vitro) environment an enhancement of reporter activity when Pkp3 was present in conjunction with ETV1.

Finally, we evaluated the impact of xPkp3 upon direct endogenous ETV1 target-genes in vivo. As shown in Figure 5D, xPkp3 knockdown in *Xenopus* embryos significantly reduced expression of established ETV1 targets involved in the dopamine transport and synthesis pathways, including dopa decarboxylase (DDC), tyrosine hydroxylase (TH), solute carrier family 18 member 2 (SLC18A2), and GTP cyclohydrolase I (GCH). These effects could be partially rescued upon the coordinate expression of ectopic ETV1. These findings continue to be consistent with Pkp3 being a positive modulator of ETV1-mediated gene regulation at promoters in vivo.

## Discussion

A significant expansion and diversification of the p120- and Pkp-catenin sub-families occurred during vertebrate evolution, resulting in proteins with distinct as well as overlapping functional activities [1], [58]. Multiple catenin roles remain unknown, but established roles include those in cadherin-based cell-cell junction function, the modulation of small-GTPases (e.g. cytoskeletal control) and gene regulation [3], [5], [32], [59]–[65]. In cells subjected to mechanical stress, the Pkp-catenins localize and function at desmosomes. Interestingly, but beyond the scope of this study, we found Pkp3 to oligomerize (possibly dimerize?) at cell-cell borders via fluorescence-lifetime imaging microscopy (data not shown). Pkps also localize to the cytoplasm or nucleus, depending on the cell type and environmental factors [25], [28], [29], [31]. While the desomosomal roles of Pkp’s have been reasonably well studied, their cytoplasmic and nuclear functions are less defined [2], [6], [20]–[22], [26], [27]. Individual catenins appear to have distinct interactions with transcription factors, even as some interactions may be shared. For example, beta-catenin and p120-catenin respectively bind to the TCF/LEF and Kaiso transcriptional repressors [32]–[34]. Intriguingly, some gene targets appear to be regulated coordinately by beta-catenin:TCF/LEF and p120-catenin:Kaiso, with upstream canonical-Wnt signals regulating the nuclear activity of both beta-catenin and p120-catenin (isoform1) [66]–[70]. Recent evidence has suggested that Pkp1 and Pkp2 may have differing roles in the nucleus, with Pkp1 binding to single-stranded DNA and Pkp2 associating with RNA polymerase III [26], [35]. It is currently unclear whether Pkp1 or 2 associate with ETV1 (or ETV5), as reported here for Pkp3. To our knowledge, in resolving Pkp3’s interaction with ETV1, we demonstrate for the first time that a Pkp-catenin functions in association with a site-specific DNA-binding transcription factor. In this regard, Pkp3’s nuclear role(s) may be analogous in concept to those previously established for catenins of the beta- and p120-subfamilies.

Our yeast two-hybrid screen using full-length Pkp3 as bait revealed multiple potential binding partners, including several gene-regulatory proteins such as ETV1 and 5. The mouse brain cDNA library used in this screen was chosen because of our earlier observations of xPkp3’s possible roles in neural development [24] and because of the low or absent expression of desmosomal components in brain that otherwise might dominate the screen and interfere with resolution of less obvious but important nuclear interactions.

We used a co-immunoprecipitation approach to confirm the Pkp3:ETV1 association under exogenous and endogenous expression contexts. The endogenous co-immunoprecipitations from fractionated mouse embryonic stem cells in Figure 1C revealed that this interaction is occurring in nuclei of this model system. In conjunction with such co-immunoprecipitations, we showed that Pkp3 localizes to both the nucleus and cytoplasm of other cell lines where ETV1 is present, in keeping with our previous observations of Pkp3 in *Xenopus* naïve ectoderm (“animal cap”) cells [24]. While requiring further investigation, Pkp3 partially re-localizes to the nucleus in the presence of exogenous ETV1 (Figure S1B). Since Pkp-catenins lack defined NLSs, it is possible that ETV1 assists with its nuclear transport. However, other good possibilities include the presence of an unrecognized NLS in Pkp3 or its assisted nuclear entry through undefined factors.

We found that the Pkp3 binding region of ETV1 (amino acids 257–335) overlaps with a previously defined inhibitory domain within the PEA3 Ets-subfamily [40], [71], [72]. Work from others has shown that when this region is bound by an antibody, the DNA binding region is unmasked and more readily binds DNA [73]. We speculate that Pkp3 binding to ETV1 may act similarly, biasing ETV1’s DNA-binding domain towards an open state to promote interaction with Ets-consensus sites and transcriptional activity.

The Pkp3:ETV1 interaction was further supported in functional assays, where ETV1 expression was able to significantly rescue the loss of neural crest markers that otherwise result upon Pkp3 knockdown. Given the model noted above, we hypothesize that the exogenous expression of ETV1 provides for a higher number of transiently open ETV1 molecules. Such open-state ETV1 would then bind to sequence-specific promoter sites to enable the partial rescue of Pkp3 knockdown effects. It is also possible that overexpressed ETV1 binds to other endogenous Pkp sub-family members, which assist in promoting or stabilizing its DNA-binding state. Indeed, preliminary results suggest that Pkp2 (but not Pkp1) enhances ETV1 activity as assessed using artificial luciferase reporters (data not shown). In any case, our findings indicate that Pkp3 can modulate ETV1 transcriptional activity as measured by both reporter gene assays and endogenous gene expression. Based upon Pkp3 depletion and other analyses (e.g. Fig. 5C), Pkp3 acts positively at established ETV1 target genes required for dopaminergic neural differentiation. This relationship might account for earlier phenotypic effects we observed in developing *Xenopus* embryos, where Pkp3 depletion led to clear perturbations of sensory neural tracts and to reductions in the responsiveness of early and tail bud stage embryos to touch [24].

With respect to the reporter assays, Pkp3’s positive effect when co-expressed with ETV1 was obvious when using the artificial (3×) Fes Ets-binding element, while a more marginal but still significant effect arose using the natural MMP-1 promoter fragment. Pkp3’s ability to activate the reporter on its own or in conjunction with exogenous ETV1 was largely eliminated upon mutation of the ETV1 consensus-binding site. This suggests, as we would predict, that Pkp3’s effects are dependent upon ETV1’s direct association with DNA. However, while requiring future investigation, Pkp3 alone activated the artificial reporter in the context of an in vitro cell-free system. While conjectural, this might arise from Pkp3’s effects upon RNA Polymerase III, as the latter has been reported to associate with Pkp2 [35].

Our results are consistent with the view that Pkp3 acts positively in association with ETV1. None-the-less, ETV1 retains significant activity upon lowering Pkp3 levels. Later work will be required to determine whether Pkp1 and Pkp2 also associate with ETV1 and thus potentially compensate in part for Pkp3 loss. Future work will also address how Pkp3-catenin confers positive gene-regulatory effects in association with ETV1, whether through potential conformational effects (above) or via the recruitment of transcriptional co-activators as is the case of beta-catenin in association with TCF/LEF (reviewed in Saito-Diaz et al., 2013 and Mosimann et al., 2009) [74], [75].

In summary, our findings identify ETV1as the first nuclear binding partner of Pkp3 and the first site-specific DNA-binding transcription factor bound to any plakophilin-catenin, to our knowledge. Given these data and ETV1’s proposed role in regulating matrix metalloproteinases during tumorigenesis [40], [76]–[78], it may prove worthwhile to test ETV1 target gene expression in cancer patients with elevated Pkp3 levels [79], [80]. Likewise, we anticipate that functional losses or gains of Pkp3-catenin might prove relevant in certain neurological contexts, given ETV1’s established roles in the dopaminergic pathway of differentiating neurons.

## Supporting Information

Figure S1 **Pkp3 localizes to nuclei of mouse embryonic stem cells and in cells exogenously expressing ETV1.** **(A)** Mouse embryonic stem cells were fractionated into cytoplasm and nuclear pools, followed by immuno-blotting as indicated. Pkp3 fractionates to both the cytoplasmic and nuclear compartments. HeLa whole-cell extract was employed as a positive control. Fractionation efficiency of mouse embryonic stem cells was monitored via immuno-blotting for alpha-tubulin (cytoplasmic marker), or for lamin A/C (nuclear marker) (mouse embryonic stem cells exhibit low lamin A/C immuno-blot reactivity as compared to HeLa cells). **(B)** Overexpression of xETV1 re-localizes a proportion of exogenous xPkp3 to the nucleus in HEK293T cells. Cells were fixed and co-immunostained for xETV1 (anti-myc, panels a and c) and xPkp3 (anti-HA, panels b and d). When Pkp3 is expressed alone (panels a and b) it localizes to the cytoplasm and cell borders. Co-expression of Pkp3 with ETV1 (panels c and d) results in increased nuclear localization of Pkp3, while still retaining its cytoplasmic localization. Representative images of a single optical section are shown, taken from a minimum of 100 cells expressing either Pkp3 alone or both constructs.
(TIF)

Figure S2 **Ectopic mitochondrial outer membrane (MOM) localization of Pkp3 was achieved by fusing a peptide sequence tag derived from human Bcl-Xl** [**46**] **, that directs the fusion product (HA-xPkp3-MOM) to the MOM, producing a distinctive punctate pattern surrounding the nucleus that is characteristic of mitochondrial localization (right panel).** An HA-Pkp3 construct lacking a MOM fusion tag (left panel), shows an entirely different localization pattern (cell-cell junctions, cytoplasmic and nuclear presence). All transfected cells observed demonstrated this patterning.
(TIF)

Figure S3 **Binding domain mapping between xPkp3 and xETV1.** **(A)** MOM-targeted xPkp3 or MOM-targeted xARVCF (negative/specificity-control catenin) was co-expressed in HeLa cells with either full-length xETV1 (FL), one of the xETV1 deletion constructs depicted in Figure 2B (CT or ΔC), or CoREST (negative/specificity-control transcription factor). Cells were then fixed and co-immunostained for ETV1 or CoREST (anti-myc, left panels) as well as MOM-targeted xPkp3 or xARVCF (anti-HA, middle panels). Combined images show the overlap of signal in yellow (merge). Representative images from a minimum of 100 cells expressing both constructs analyzed are shown. **(B)** Single optical section of cell co-expressing MOM-targeted xPkp3 along with the construct xETV1-CT as described above in Fig. S3A. Subpanels (a′, a′′, a′′′) are shown prior to intensity adjustment. Subpanels (b′, b′′, b′′′) are from the same image as (a′, a′′, a′′′), but the intensity has been increased to show the absence of ETV1-CT signal at sites of MOM-targeted xPkp3 localization.
(TIF)
