# Supplementary figures and images for: Plakophilin-3 Catenin Associates with the ETV1/ER81 Transcription Factor to Positively Modulate Gene Activity
Source: PLoS One. 2014 Jan 27;9(1):e86784. doi: 10.1371/journal.pone.0086784 (PMC3903613; doi:10.1371/journal.pone.0086784)

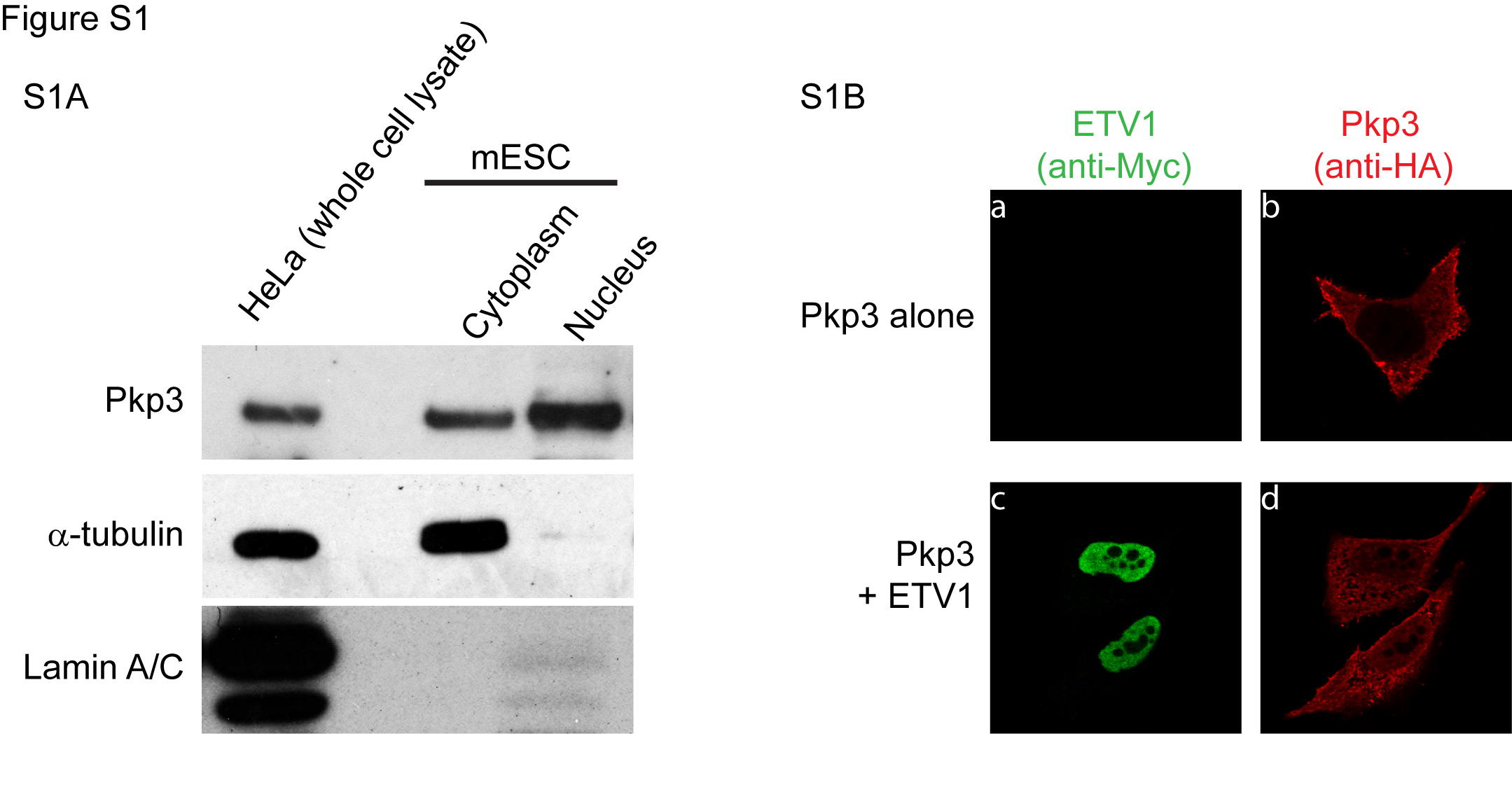

Supplement: Figure S1 — Pkp3 localizes to nuclei of mouse embryonic stem cells and in cells exogenously expressing ETV1. (A) Mouse embryonic stem cells were fractionated into cytoplasm and nuclear pools, followed by immuno-blotting as indicated. Pkp3 fractionates to both the cytoplasmic and nuclear compartments. HeLa whole-cell extract was employed as a positive control. Fractionation efficiency of mouse embryonic stem cells was monitored via immuno-blotting for alpha-tubulin (cytoplasmic marker), or for lamin A/C (nuclear marker) (mouse embryonic stem cells exhibit low lamin A/C immuno-blot reactivity as compared to HeLa cells). (B) Overexpression of xETV1 re-localizes a proportion of exogenous xPkp3 to the nucleus in HEK293T cells. Cells were fixed and co-immunostained for xETV1 (anti-myc, panels a and c) and xPkp3 (anti-HA, panels b and d). When Pkp3 is expressed alone (panels a and b) it localizes to the cytoplasm and cell borders. Co-expression of Pkp3 with ETV1 (panels c and d) results in increased nuclear localization of Pkp3, while still retaining its cytoplasmic localization. Representative images of a single optical section are shown, taken from a minimum of 100 cells expressing either Pkp3 alone or both constructs. (TIF) [file pone.0086784.s001.tif]

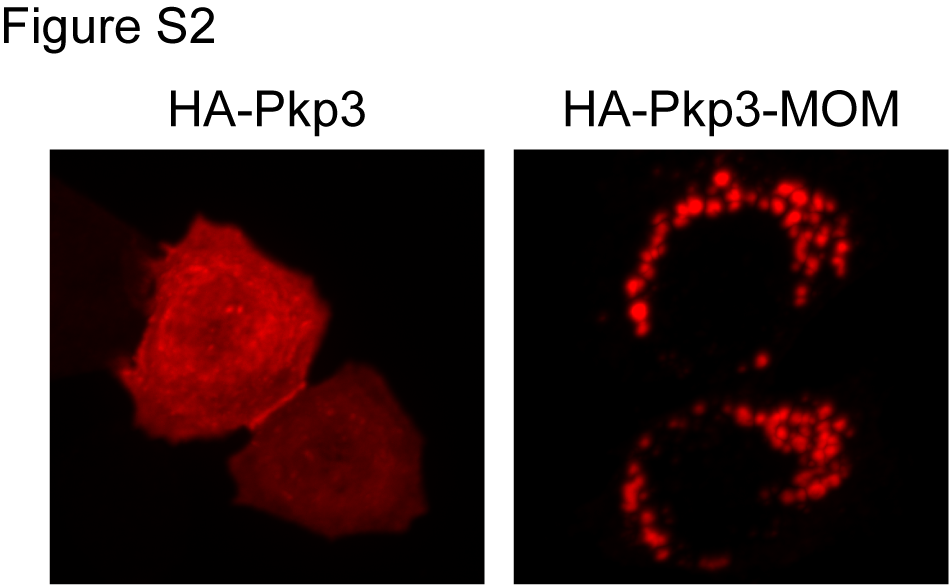

Supplement: Figure S2 — Ectopic mitochondrial outer membrane (MOM) localization of Pkp3 was achieved by fusing a peptide sequence tag derived from human Bcl-Xl [46] , that directs the fusion product (HA-xPkp3-MOM) to the MOM, producing a distinctive punctate pattern surrounding the nucleus that is characteristic of mitochondrial localization (right panel). An HA-Pkp3 construct lacking a MOM fusion tag (left panel), shows an entirely different localization pattern (cell-cell junctions, cytoplasmic and nuclear presence). All transfected cells observed demonstrated this patterning. (TIF) [file pone.0086784.s002.tif]

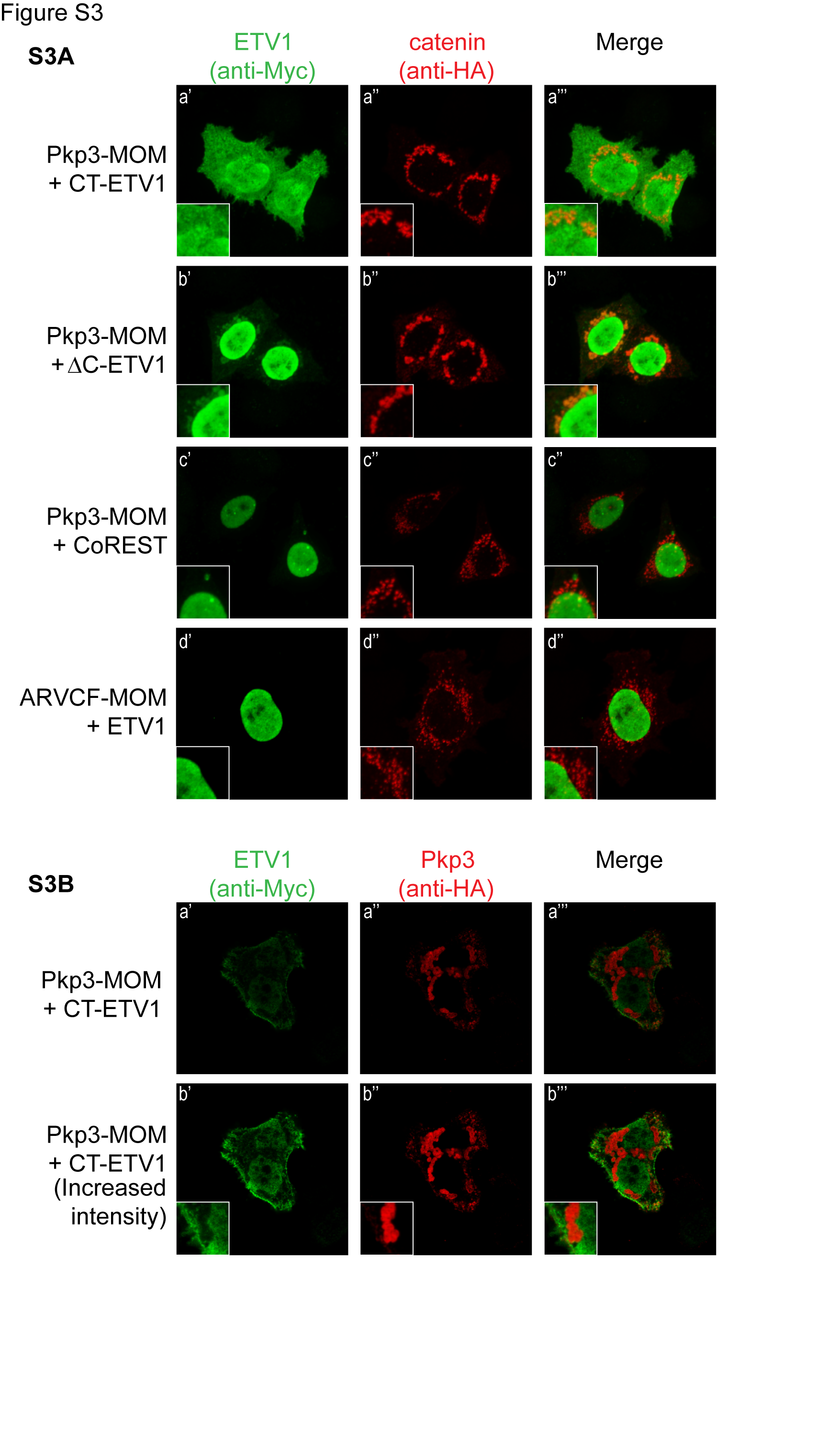

Supplement: Figure S3 — Binding domain mapping between xPkp3 and xETV1. (A) MOM-targeted xPkp3 or MOM-targeted xARVCF (negative/specificity-control catenin) was co-expressed in HeLa cells with either full-length xETV1 (FL), one of the xETV1 deletion constructs depicted in Figure 2B (CT or ΔC), or CoREST (negative/specificity-control transcription factor). Cells were then fixed and co-immunostained for ETV1 or CoREST (anti-myc, left panels) as well as MOM-targeted xPkp3 or xARVCF (anti-HA, middle panels). Combined images show the overlap of signal in yellow (merge). Representative images from a minimum of 100 cells expressing both constructs analyzed are shown. (B) Single optical section of cell co-expressing MOM-targeted xPkp3 along with the construct xETV1-CT as described above in Fig. S3A. Subpanels (a′, a′′, a′′′) are shown prior to intensity adjustment. Subpanels (b′, b′′, b′′′) are from the same image as (a′, a′′, a′′′), but the intensity has been increased to show the absence of ETV1-CT signal at sites of MOM-targeted xPkp3 localization. (TIF) [file pone.0086784.s003.tif]
